# Detection of Black Plastics in the Middle Infrared Spectrum (MIR) Using Photon Up-Conversion Technique for Polymer Recycling Purposes

**DOI:** 10.3390/polym9090435

**Published:** 2017-09-08

**Authors:** Wolfgang Becker, Kerstin Sachsenheimer, Melanie Klemenz

**Affiliations:** Fraunhofer Institut fuer Chemische Technologie (ICT), P.O. Box 12 40, 76327 Pfinztal, Germany; Kerstin.Sachsenheimer@ict.fraunhofer.de (K.S.); Melanie.Klemenz@ict.fraunhofer.de (M.K.)

**Keywords:** black plastic recycling, photon up conversion, MIR spectroscopy

## Abstract

The identification of black polymers which contain about 0.5 to 3 mass percent soot or black master batch is still an essential problem in recycling sorting processes. Near infrared spectroscopy (NIRS) of non-black polymers offers a reliable and fast identification, and is therefore suitable for industrial application. NIRS is consequently widely used in polymer sorting plants. However, this method cannot be used for black polymers because small amounts of carbon black or soot absorb all light in the NIR spectral region. Spectroscopy in the mid infrared spectral region (MIR) offers a possibility to identify black polymers. MIR spectral measurements carried out with Fourier-transform infrared spectrometers (FTIR) are not fast enough to meet economic requirements in sorting plants. By contrast, spectrometer systems based on the photon up-conversion technique are fast and sensitive enough and can be applied to sort black polymer parts. Such a system is able to measure several thousand spectra per second hence is suitable for industrial applications. The results of spectral measurements of black polymers are presented.

## 1. Introduction

Black polymers are widely used in consumer products such as electronic household goods, automobile components or IT goods. The black color is achieved by adding 0.5 to 3 mass percent soot or black master batch in the polymer melt during the extrusion process. The recycling and reuse of these materials is of substantial value and reliable methods for the identification of black polymers are still under development. For industrial applications identification techniques have to be economical, i.e., fast enough, reliable with respect to long term stability, safe and easy to operate. Only methods fulfilling these requirements are suitable for large scale recycling applications. For non-black plastic materials, e.g., household goods, NIRS is the predominant spectroscopic technology. However, because of the soot contained in black plastics, NIRS cannot be used, as all light is absorbed, rendering material recognition and classification impossible. MIR spectroscopy is an option to measure and characterize black plastics [1].

Black polymers represent a much wider variety of materials than do household plastic waste since they are mostly used for technical applications with special requirements. Different additives and filler materials, which are added in order to achieve specific properties of the plastics, complicate the identification, since spectra of the same kind of plastic can vary dramatically if different types or amounts of additives, e.g., flame retardants, fibers, or soot are contained in the plastic parts. Lacquer films on the plastic part surface even prevent any spectral identification and have to be removed before measurement. Interest in characterizing black polymers has led to a wide range of IR-techniques e.g., attenuated total reflection (ATR) [2,3] infrared transmission [3,4] emission spectroscopy [5], and photoacoustic spectroscopy [6]. In several publications the use of reflectance measurements was demonstrated for characterizing soot filled polymers [2,7,8,9,10].

Photon up-conversion is a possibility to combine the infrared and the NIR spectral range. Photons are transformed from the IR spectral range into the near infrared spectral range, in which fast silicon detectors can be used. Up-conversion is carried out by adding the infrared photon energy to that of the photons of a Neodym-doped Yttrium–Vanadat laser to produce the sum frequency, resulting in a higher energy than the band gap of silicon material.

This concept offers the possibility to measure plastic parts in the infrared range and the up-converted light can be evaluated with conventional silicon detector technology [11,12,13]. In the present paper, the reflection spectra of some technical plastics are shown and compared with conventional Fourier transform spectrometer measurements to demonstrate the potential of up-conversion spectroscopy for plastic sorting applications.

The system was tested and measurements were performed at the Fraunhofer-Institut fuer Chemische Technologie (Fh-ICT). The spectrometer system based on photon up-conversion was developed and constructed at the Fraunhofer-Institut fuer Physikalische Messtechnik (Fh-IPM).

### 1.1. Spectral Identification of Plastic Parts

The main spectral method for industrial plastic identification is near infrared spectroscopy.

Rugged detector systems based on indium gallium arsenide (InGaAs) material can be applied which are sensitive and fast enough for sorting applications. The dispersive spectrometer systems possess no moveable parts and are therefore insensitive to mechanical vibrations and can be protected against dust exposure. Sophisticated systems reach a measurement rate up to several ten thousand spectra per second which is needed for the economic running of a sorting facility.

The NIR spectrum expands the wavelength range from 780 to 2500 nm which corresponds to a wavenumber range from about 12,800 to 4000 cm^−1^. The NIR range is often subdivided into two parts because different detectors are used. In the short near infrared (SNIR) region, with wavelengths from 780 to about 1050 nm, silicon based sensors are mainly used, while in the “classical” near infrared range, from 1050 to 2500 nm wavelength, InGaAs sensors predominate. The NIR range is dominated by overtone and combination bands of C–H, N–H, O–H, and C–O functional groups. The identification of plastics is mainly based on the stretching vibration modes of CH, CH_2_ and CH_3_ groups between about 1.1 and 1.25 μm, which correspond to the second overtone, the first overtone ranging from 1.65 and 1.7 μm, and the combination bands, respectively [14]. The main limitation of plastic identification with NIR spectroscopy is that black plastics cannot be detected. The soot they contain absorbs all light in the NIR range and no detectable signal can be evaluated for material separation.

**Figure 1:**
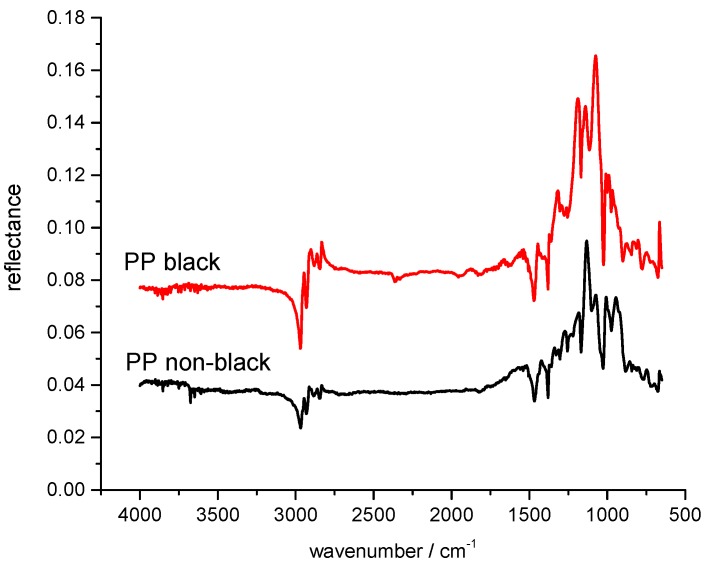
Comparison of IR spectra of the surface reflectance of a black and a non-black polypropylene (PP) plastic part.

In the MIR spectral region from 2.5 to about 16 µm wavelength, which corresponds a wavenumber range from 4000 to about 600 cm^−1^, the different kinds of plastic material show additional vibrational modes like deformation, rocking, and twisting modes due to their molecular structure. Beside the C–H group, other molecules like O–H, N–H and O–C also contribute with their fundamental vibrations to the spectral features. The various molecular groups with their different vibrational modes generate a unique spectrum of each polymer in the spectral range between 2500 and 600 cm^−1^, which allow a definite identification. This spectral range is therefore called the fingerprint region, and MIR spectroscopy is the predominant analytical method for polymer investigation and characterization. Another important advantage of the MIR spectral range is that black polymers can be measured. Reflectance spectra can be measured and allow identification of the black polymers. This is shown in Figure 1 where the reflectance spectra of a black polypropylene (PP) polymer part and a non-black part are compared. The spectra show the main spectral characteristics, and the filler material has no obvious influence on the optical properties of the polymers. MIR is therefore a suitable analytical method for the identification of black polymers.

### 1.2. Non-Linear Photon Up Conversion 

In the photon up conversion process infrared photons are converted to higher energy photons in the NIR range i.e., in the silicon spectral range by sum frequency. An MIR photon with energy ω*_MIR_* interacts with a pumping laser photon ω*_LS_* in a non-linear medium to generate a third photon with energy ω*_SF_*.

(1) $$\omega_{MIR}+\;\omega_{LS}=\;\omega_{SF}$$

Using this mechanism the high conversion of signals has long been known [15]. By using periodically polarized non-linear crystals made of lithium niobate oxide (PPLN), and by the proposal to carry out the high conversion directly in the laser resonator, whereby the laser power is amplified, a conversion rate of more than 10% can be achieved. In addition to the energy conservation according to Equation (1), a phase matching of the light wave in the crystal must also be present. This can be formulated in the k-space with k-vectors and in the one dimensional case it corresponds to Equation (2).

(2) $$\frac{1}{\Lambda_{\mathrm{PP}}}+\;\frac{n_{LS}}{\lambda_{LS}}+\frac{n_{MIR}}{\lambda_{MIR}}=\frac{n_{SF}}{\lambda_{SF}}$$

Λ_PP_ is the polarization period of the conversion crystal and the different λ*_i_* are the wavelengths of the interacting waves. The *n_i_* are the refractive indexes of the media at the respective wavelength. The refractive index of the crystal is temperature dependent. By choosing a temperature and the polarization period of the crystal the wavelength range of the converter is determined. More details and applications of photon up conversion can be found in [16].

## 2. Experiments

**Figure 2:**
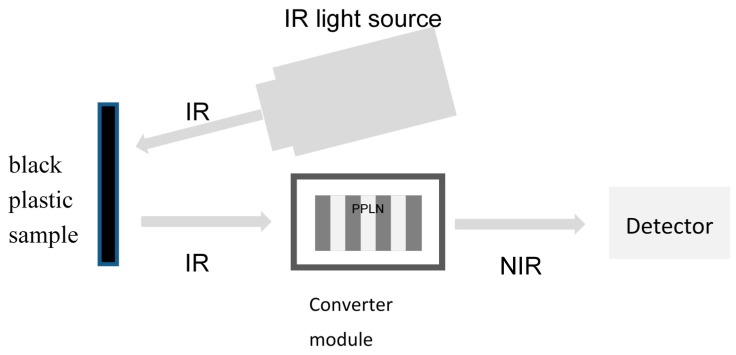
Schematic drawing of the measuring setup for the measurement of black plastics in reflection with the photon up conversion set-up.

The principal system setup (Fraunhofer Institut für Physikalische Messtechnik IPM, Freiburg, Germany) which was used to measure the plastic samples is shown in Figure 2. The IR light source was a black body radiator with a temperature of 900 °C. The sample parts were plastic plates with a thickness of 5 mm. The samples were not electrically conductive. This distinction is important because black plastic samples which are electrically conductive also absorb the entire radiation in the MIR spectral range and could not be characterized. The sample was irradiated with the black body and the reflected light passed the converter module with the PPLN crystal. The generated NIR signal was measured with a single photon avalanche diode area sensor. The sensor used was also a development within the framework of the research project LITRAN within the Fraunhofer Gesellschaft, carried out by the Fraunhofer-Institut für intergrierte Schaltungen (Fh-IIS). The measurement time was 0.3–0.5 s for one sample. The reference measurement *I*_R_ was carried out with an uncoated aluminum mirror. The reflectance was calculated according to Equation (3).

(3) $$R=\;\frac{I_{S}-\;I_{N}}{I_{R}-I_{N}}$$

*I*_S_ is the sample intensity spectrum and *I*_R_ is intensity of the reference.

*I*_N_ is the wavelength dependent noise and the dark current spectrum.

The poling period Λ_PP_ of the PPLN crystal was Λ_PP_ = 21.34 µm and the temperature of the PPLN crystal was 50.02 °C for all measurements. The measured NIR spectra were then assigned to wavelengths in the MIR.

In the following section, the measurements with the photon up-conversion system are referred to as LITRAN (from Light Transformation). This will simplify the presentation.

## 3. Results

**Figure 3:**
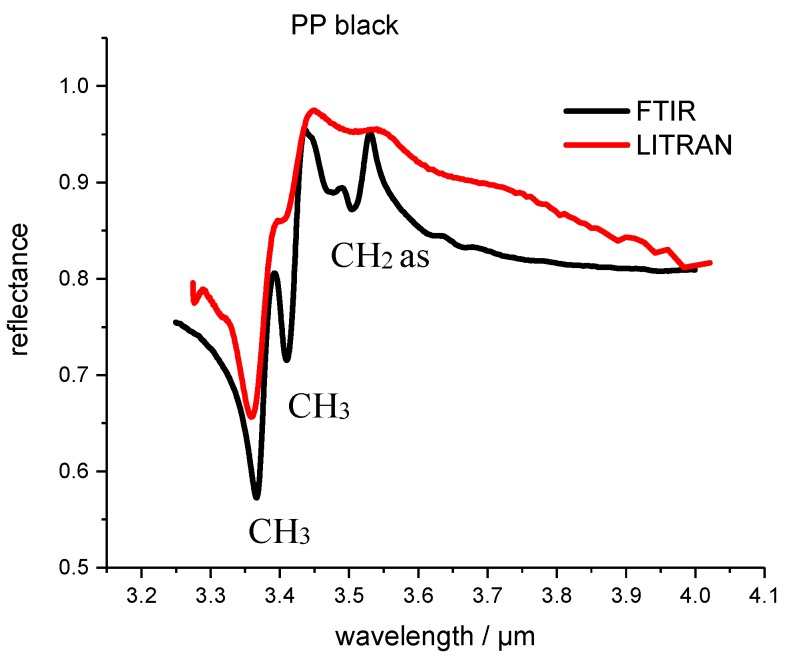
Fourier-transform infrared spectrometers (FTIR) and the measurements with the photon up-conversion system are referred to as LITRAN (from Light Transformation) of the reflectance spectrum of PP black polymer. LITRAN is the spectrum measured with the up conversion system.

A polypropylene (PP) black plastic sample was measured with the photon up conversion system (Fraunhofer Institut für Physikalische Messtechnik IPM,) as shown in Figure 2. The temperature of the conversion crystal *T*_PPLN_ was 50.02 °C and the poling period Λ_PP_ was 21.34 µm. Thus, the spectral range was determined from 3.2 to 4.15 µm wavelength. Only one measurement was taken with no accumulations. To have a comparison and to evaluate the quality of the measured spectra of the LITRAN system, the plastic samples were also measured with a commercial FTIR spectrometer (Fraunhofer Institut für Physikalische Messtechnik IPM). The results of the spectral measurements and the comparison are shown in Figure 3.

The FTIR spectra were measured with a spectral resolution of 4 cm^−1^. Functional groups of the PP polymer are assigned to the corresponding reflection peaks. By comparing the two spectra one can see that the spectral resolution of the LITRAN system is lower compared to the FTIR measurements. The reflection peaks are wider but present and can be used for the recognition and differentiation of other types of plastic.

**Figure 4:**
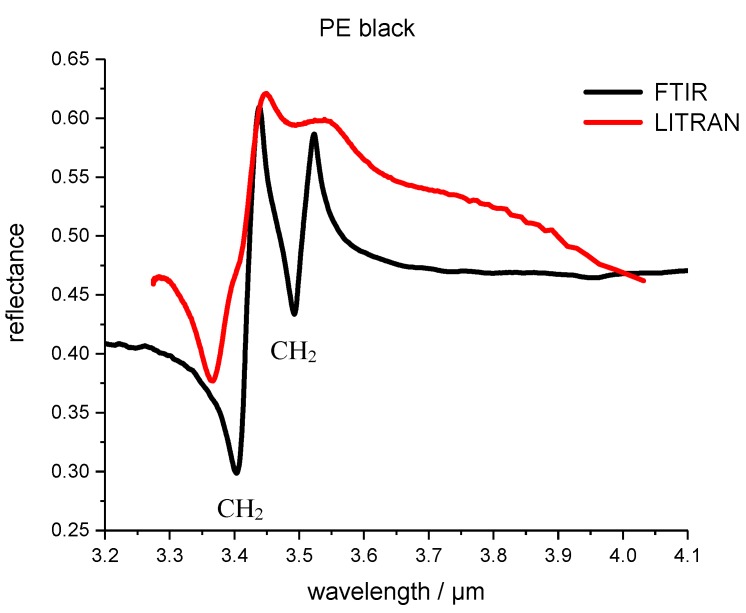
FTIR and LITRAN reflectance spectrum of polyethylene (PE) black polymer. LITRAN is the spectrum measured with the up conversion system.

Figure 4 shows the FTIR and LITRAN reflectance spectra of black PE (polyethylene). The reflection peaks are wider as in the case of the LITRAN PP spectrum. In addition, the PE measurements also show a wavelength shift compared to the FTIR spectrum of the PE sample and there is an additional shoulder in the spectrum at around 3.4 µm. The cause for this might be that the entrance angle and phase matching in the PPLN crystal varied. This means the reason for these features are caused by the measurement set-up and has nothing to do with some material properties.

**Figure 5:**
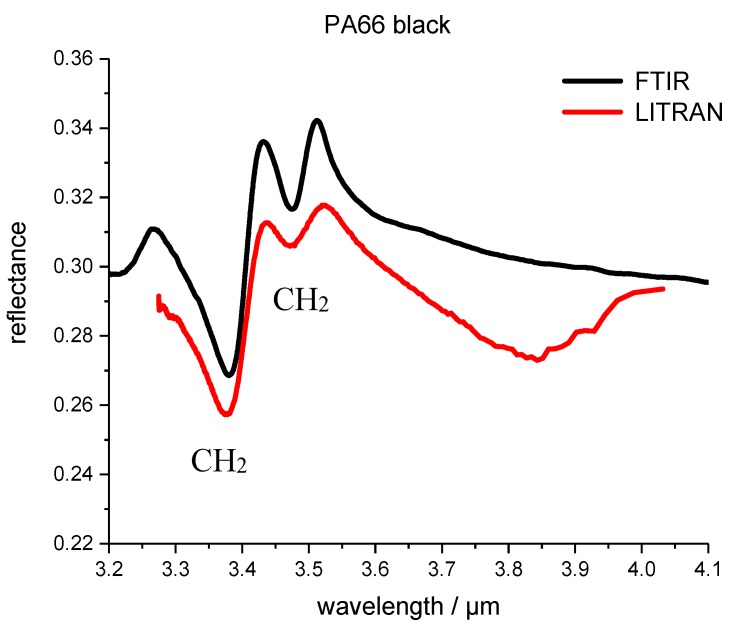
FTIR and LITRAN reflectance spectrum of polyamide 66 (PA 66) black polymer. LITRAN is the spectrum measured with the up conversion system.

Figure 5 shows the FTIR and LITRAN spectrum of PA66 (polyamide 66). As in all measurements of the plastic samples, the spectra in the wavelength range from 3.6 µm to approximately 4 µm show a very different curvature. In this spectral range the sensitivity and accuracy of the LITRAN system decreases, leading to this spectral behavior.

### 3.1. Principal Component Analysis (PCA)

PCA is an unsupervised learning method. There are no targets, but attempts are made to extract similarities and differences from the measured data—the so-called characteristics. In the case of the plastic samples, these characteristics are the measured spectra. The measured plastic spectra are first combined into a data matrix *M*. In the PCA, the original data matrix *X* is now approached by two smaller matrices *T* and *L*, which are referred to as the score matrix *T* and the loading matrix *L*. The columns in the loading matrix are also called principle components PCs and they are determined to be mutually orthogonal. Every loading or principle component describes a specific amount of the contained variance of the data matrix *M* and each main component describes a maximum of variance that has not yet been achieved by the previous ones. There are different methods of decomposing the data matrix *M* into the submatrices *T* and *L*. One of these is the singular value decomposition (SVN), which uses the maximum variance in the measured data as a criterion. The score indicates how much of a specific loading is contained in the measured data. The PCA can be formulated as:

(4) $$M=T\;L^{t}$$

For the numerical determination of the matrices *T* and *L*, the NIPALS algorithm is often used. The PCA evaluation of the available data was carried out with the Unscrambler^®^ software package from CAMO version 10.X (Oslo, Norway). More information about PCA can be found in [17,18]. The calculation of the PCA evaluation took only a few seconds. For processing purposes several thousand evaluations per second can be done.

### 3.2. Distinguishing Black Plastic

**Figure 6:**
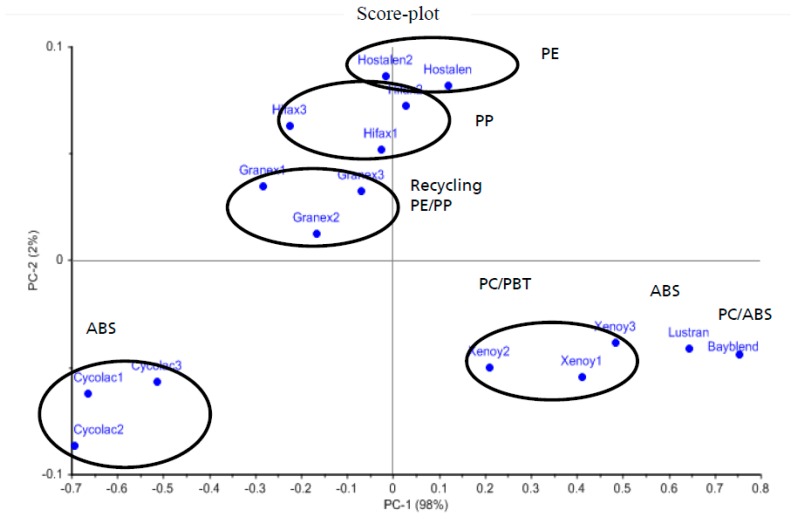
PC1–PC2 plot of the scores from principle component analysis (PCA) evaluation of the black plastic samples. Each dot in the plot in Figure 6 represents a complete spectrum.

In order to examine whether the LITRAN system can be used to distinguish between different plastic materials, several commercial plastics were measured with the LITRAN system in the wavelength range of 3.2 to 3.6 µm, which is determined by the poling period and crystal temperature and subjected to a principle component analysis (PCA). The score plot of the measurements is shown in Figure 6.

The plastic samples are bulk plastics such as PP and PE and technical plastics such as ABS (acrylonitrile–butadiene–styrene) and PBT (polybutylenterephthalate), which are used in the automotive and electrical industries. There are also some blends with PC (polycarbonate). All measured black plastic samples are commercially available and are used for industrial goods. The commercial names are each indicated in the framing of the so called clusters. At each cluster the type of plastic is shown. The two principle components PC1 (98%) and PC2 (2%) contain 100% of the spectral data.

The two dimensional score plot shows the different clusters representing the spectra of the plastic samples. PE (Hostalen) and PP (Hifax) are all in the positive part of the axis of component PC2. Both plastics are thermoplastics. Granex is a mixture of both types of recycled PP and PE, so the Granex cluster is positioned near the PE and PP cluster. Since the Granex cluster is closer to the PP cluster, one can also conclude that the Granex mixture has a larger share of PP polymer.

Cycolac and Lustran are ABS plastics from two different manufacturers. As a result, the composition of this blend also varies and there are spectral differences. These two clusters are consequently far apart in the PCA plot. This makes it possible to identify ABS plastics from different manufacturers and sort them accordingly. This can be decisive for the regranulation of these plastics because the mixing of ABS from different sources and thus different compositions can lead to a worsening of the quality of the regranulated material.

The same is valid for the PC/ABS and PC/PBT blends. These samples have different compositions compared, for example, to ABS or pure PBT, and consequently possess different spectral properties in the MIR. The t-score and the cluster are well separated from the other samples, indicating that the black plastic samples can be separated and characterized by the LITRAN system or photon up conversion.

**Figure 7:**
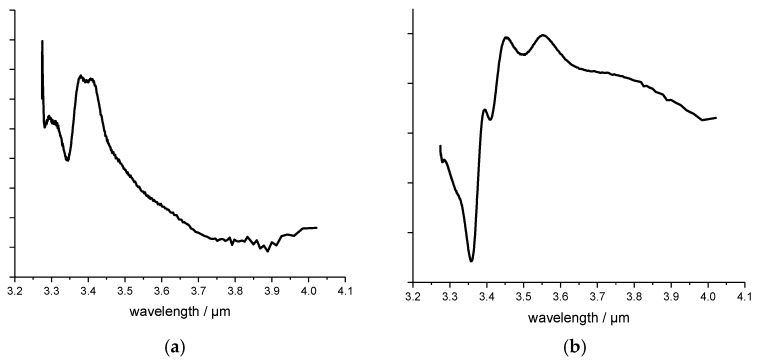
The PCA evaluation of black plastic samples (**a**) PC1; (**b**) PC2.

The principle components PC1 and PC2 of the PCA for the black plastic samples are shown in Figure 7a,b respectively. The principle components indicate the main spectral information contained in each spectrum. The first PC contains most of the spectral variances and the second PC indicates the second highest percentage of variance and so on. The scores of the samples in the score plot again indicate how much of the spectral information is contained in the measured spectra. This makes it possible to distinguish identical or similar plastic samples or highly differentiated samples from one another, and thus to classify them into plastic varieties. Looking at the measurements of the black plastic samples in the score plot in Figure 6, one can see that the samples containing ABS, PC, or PBT are arranged along the PC1. From this it can be concluded that the PC1 represents the characteristics of these plastic grades. It is noteworthy that the ABS sample with the mark name Cycolac and the ABS sample Lustran are far apart. The reason for this is that these plastics come from different manufacturers and therefore partly different additives have been added or the blend composition varies. PE and PP samples have therefore a sore value at PC1 near zero. Looking at the position of the scores of the samples with respect to PC2, it can be seen that the samples PE and PP have positive values and the scores of other samples are negative. This means that with the help of the PC2 the thermoplastic samples can be distinguished from the non-thermoplastic ones.

## 4. Conclusions

As shown, MIR spectroscopy is an efficient method for spectrally characterizing black plastic parts and assigning them to the corresponding polymer types. Based on measurements of the reflectivity in the spectral range from 3.2 to about 3.6 μm with the LITRAN system, in addition to pure plastic grades, blends can also be detected and even assigned to different manufacturers. In the case of re-granulates, for example, the proportions of the base material can be determined in principle according to corresponding calibration models. These are fundamental prerequisites for assuring accurate sorting and for achieving a high grade of recycled black plastic parts. The LITRAN system provides the prerequisite for the economical sorting of black plastics, since measuring rates of several thousand measurements per second can be achieved. As a result, sorting systems with a sufficient mass throughput can be implemented. In the case of engineering plastics, the determination of possible polymer additives and polymer mixtures is also important. Many engineering plastics contain, for example, flame retardants, some of which are prohibited and have to be sorted out before the re-granulation. The spectral range of the LITRAN system can be adapted to detect these substances.

The LITRAN system also offers the possibility to expand existing sorting systems with NIR sorting into the MIR range.

Because the absorption bands or signals are 10 to 100 times stronger in the MIR range than in the NIR range, hence the sorting of normal non-black household plastics can also be carried out with the LITRAN system. However, as a prerequisite, the measuring rate of the LITRAN system would have to be able to perform several 10,000 to 100,000 measurements per second. Optical components and the measurement setup of the LITRAN system are in principle capable of extending the application range of photon up conversion to other industrial applications.
